# A systematic review of primary Sjögren’s syndrome in male and paediatric populations

**DOI:** 10.1007/s10067-017-3745-z

**Published:** 2017-07-22

**Authors:** Simrun Virdee, James Greenan-Barrett, Coziana Ciurtin

**Affiliations:** 1 University College London Medical School, London, UK; 2 Department of Rheumatology, University College London, 250 Euston Road, London, NW1 2PG UK

**Keywords:** Primary Sjögren’s syndrome, Male patients, Children, Systematic review

## Abstract

Primary Sjögren’s syndrome (pSS) is a chronic multisystem autoimmune rheumatic disease characterised by female predominance. Although the disease is rare in the male and paediatric populations, it has been suggested that it may have a different disease phenotype, which has not been investigated before using a systematic approach. A systematic literature search of PubMed databases (updated to December 2016) was performed to identify all published data on the epidemiological, clinical and laboratory manifestations of pSS in the male and paediatric populations. The literature search of the male and paediatric pSS studies identified 2025 and 186 citations, respectively, out of which 7 and 5 fulfilled our inclusion criteria and were analysed further. The range of age at disease onset was 9.4–10.7 years for children and 39.4–56.9 years at diagnosis for male patients. We identified a prevalence of extra-glandular manifestations between 52.6–92.3% in the male population and 50.0–84.6% in children, while abnormal sialometry was only reported in the paediatric population, with a prevalence between 71.4 and 81.8%. There was a significant variation of positive serological markers, with anti-Ro antibodies reported between 15.7–75.0% and 36.4–84.6%, and anti-La antibodies between 5.6–51.7% and 27.3–65.4%, in the male and paediatric populations, respectively. The characteristics of pSS in the male and paediatric populations varied according to different studies. When compared to data available from pSS adult populations, children diagnosed with pSS reported less dryness and had a higher prevalence of parotitis, lymphadenopathy and systemic symptoms and male patients were younger at the time of diagnosis. This systematic review contributes to a better understanding of the epidemiology of pSS in rare populations. Large longitudinal cohort studies comparing male with female patients and adult with paediatric patients are needed.

**Electronic supplementary material:**

The online version of this article (doi:10.1007/s10067-017-3745-z) contains supplementary material, which is available to authorized users.

## Introduction

Primary Sjögren’s syndrome (pSS) is a chronic multisystem autoimmune rheumatic disease, associated in the majority of cases with infiltration of exocrine glands, particularly the salivary and lacrimal glands, by lymphocytes and plasma cells, resulting in xerostomia and xerophthalmia. pSS is a heterogeneous disease; clinical manifestations range from localised glandular disease to complex and even life-threatening systemic features affecting the neurological, renal, musculoskeletal, dermatological, haematological, and pulmonary systems. This autoimmune rheumatic disease is 9–20 times more common in females than males according to different studies [1–4], and although commonest between the ages of 30 and 60, it is also described in younger and older age groups [5]. There is a similar female predominance in the paediatric population (F:M = 5–7:1) [6, 7]. The prevalence of pSS in the general population ranged in Greece from 0.09 to 0.23% [4, 8], in the UK from 0.14 to 1.60% [9] and in Slovenia was estimated at 0.60% [10].

In 2016, newly updated classification criteria were published by the American College of Rheumatology/European League Against Rheumatism [11, 12]. The proposed classification criteria weigh 5 criteria items with a score of either 1 or 3 and a total score of at least 4/9, in the absence of any exclusion criteria, is required for a diagnosis. These weighted criteria improve on the previous American-European Consensus Group (AECG) and American College of Rheumatology (ACR) classification criteria by ensuring that a positive anti-SSA/Ro or salivary gland biopsy is required for a diagnosis. Along with other changes, in the absence of anti-SSA/Ro, anti-SSB/La is no longer considered a criteria item.

## Methods

### Search strategy

We performed a systematic literature search of PubMed, covering papers published between January 1984 and December 2016. In search of the articles on the male population with Sjögren’s syndrome, we used the following MeSH terms: ‘Sjögren’s syndrome’ and ‘men’ OR ‘male’. To identify relevant publications on the paediatric population, we used ‘Sjögren’s syndrome’ and ‘child’ OR ‘paediatrics’ as MeSH terms. The automatically selected papers were manually screened by reading the titles and abstracts. Both searches were limited to human studies and English language publications but no limitations were based on the country of origin or ethnicity of patients. Following the selection of articles that met the inclusion criteria, their references were reviewed for any relevant papers that had been missed by the PubMed search, and relevant studies were added to the final analysis.

### Study selection

The study selection was carried out independently by two authors (SV and JGB) based on the inclusion criteria mentioned above, and subsequently compared—any discrepancies were discussed with the third author (CC) and a consensus was reached. The general exclusion criteria applied by authors were the following: incorrect patient groups (female cohort, secondary Sjӧgren’s syndrome, patients selected based on specific comorbidities), incorrect study types (case reports, interventional studies, editorials, commentaries, surveys and questionnaires), studies that analysed therapeutics, quality of life or cost-benefit analysis and genetic, laboratory or pathology studies. The full texts of the remaining articles were read and the above criteria were further applied.

**Fig. 1:**
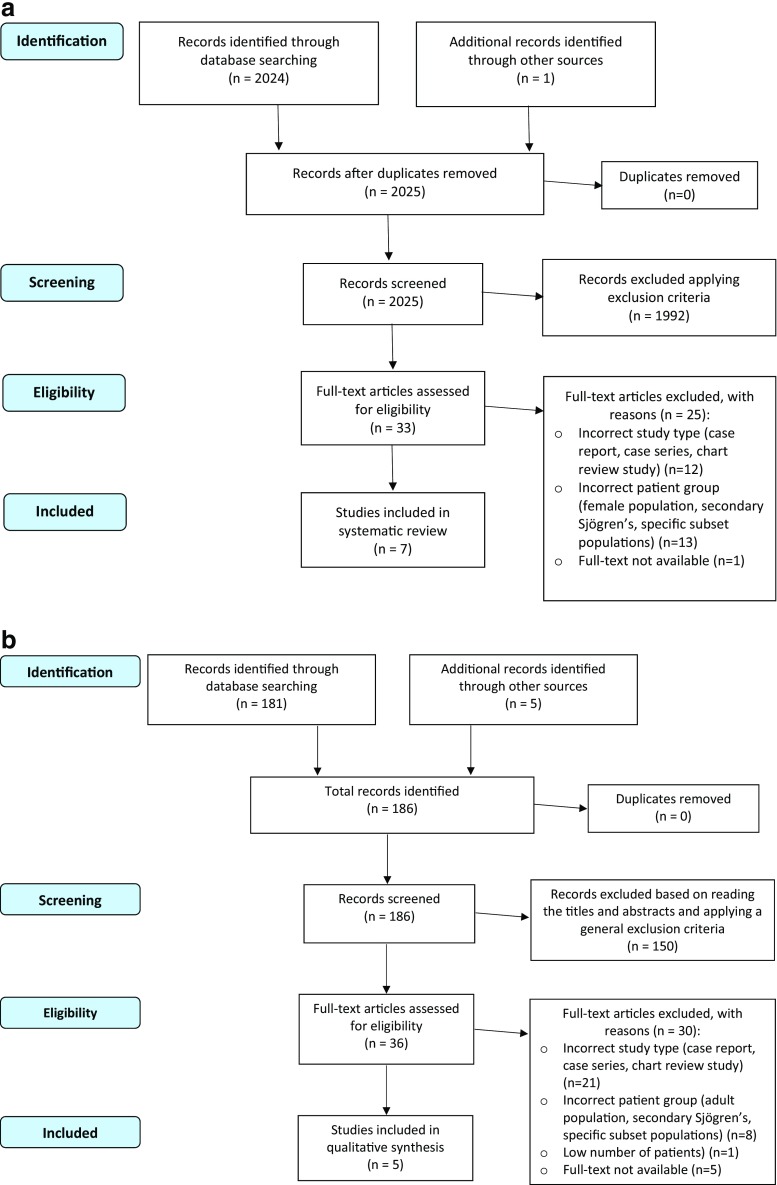
**a** Flowchart of study selection for papers referring to the pSS male population. **b** Flowchart of study selection for papers referring to pSS paediatric populations

The data from the eligible articles was then extracted and analysed. The study selection process is detailed in Fig. 1. One article (retrieved as a reference in another paper) was excluded from the final analysis, as despite our attempts to contact the author, the full text could not be retrieved [13].

### Data extraction

We extracted data on epidemiological (study type, number of patients, country of origin, etc.), clinical and laboratory features and presented it in four tables. Table 1 details the epidemiological data on both male and paediatric populations. In Table 2, we included information related to the clinical features associated with pSS in both populations. The laboratory data were organised into two tables (Tables 3 and 4) as the data set analysed differed between the male and paediatric population. Any data reported as percentages were converted to actual number of patients to allow us to calculate ranges of data across all the studies analysed.

**Table 1:** Papers included in the systematic review

References	Study type	Country of origin	N of pSS patients	Criteria for inclusion of patients	Mean age	Female: male ratio	JBI criteria fulfilled (out of 9)
Male studies							
Molina et al. [16]	Cross-sectional study	US	36	Personal^a^	50 (at diagnosis)	1.91:1	5
Anaya et al. [17]	Case-control study	US	13	ECSG	39.4 (at diagnosis)	2:1	4
Horvath et al. [18]	Cross-sectional study	Hungary	60	AECG	56.18 (at study)	7:1	6
					47.83 (at diagnosis)		
Gondran et al. [19]	Consecutive case series—multicentre study	France	42	AECG	56.9 (at diagnosis)	10.1:1	6
Cervera et al. [20]	Consecutive case series study	Spain	19	ECSG	55 (at onset)	10.7:1	5
Diaz-Lopez et al. [21]	Cross-sectional study	Spain	28	ECSG	63.5 (at study)	18.6:1	6
Drosos et al. [22]	Case selection for male patients, consecutive case series for female patients	Greece	12	ECSG	45.3 (at onset)	2.5:1	5
Paediatric studies							
Yokogowa et al. [23]	Retrospective case-control study	USA	26	Personal^b^	12.4 (at diagnosis)	24:2	4
Cimaz et al. [24]	Multicentre case series study	Italy	40	Personal^c^	10.6 (at onset)	35:5	7
Drosos et al. [22]	Case selection for paediatric patients, consecutive case series for female patients	Greece	13	ECSG	9.4 (at onset)	11:2	5
Stiller et al. [25]	Retrospective comparative study	Berlin	11	EULAR	9.5 (at onset)	10:1	5
Tomiita et al. [26]	Cross-sectional epidemiological study	Japan	42	Personal^d^	10.7 (at onset)	–	6

*AECG* American-European Consensus Group, *ECSG* European Community Study Group, *EULAR* European League Against Rheumatism, *JBI* Joanna Briggs Institute

^a^Sicca syndrome + 2 of the following: (1) abnormal Schirmer’s test, (2) KCS by Rose Bengal dye test and (3) positive biopsy (Greenspan Grade 3 or 4)

^b^Diagnosis of pSS by a paediatric rheumatologist and followed for a minimum of 1 year with no subsequent alternative diagnosis

^c^Diagnosis of pSS by referring clinician + onset <16 years

^d^Histopathological changes in salivary gland (>3 on Greenspan scale)

**Table 2:** Glandular and extra-glandular features of pSS in male and paediatric populations

	Glandular					Extra-glandular (EG)							
References	Dry eyes *N* (%)	Dry mouth *N* (%)	Parotitis *N* (%)	Schirmer^a^ *N* (%)	Any sicca N (%)	Any EG N (%)	Joint Involvement *N* (%)	Raynaud’s *N* (%)	Pulmonary *N* (%)	Neurological *N* (%)	Renal *N* (%)	Lymphadenopathy *N* (%)	Other *N* (%)
Male studies													
Molina et al. [16]	14 (38.9)	–	10 (27.8)	19 (52.8)	36 (100)^b^	29 (80.6)	28 (77.8)	4 (11.1)	1 (2.8)	14 (38.9)	IgA nephropathy—1 (2.8)	2 (5.6)	Vasculitis—9 (25.0), splenomegaly—3 (8.3), hepatomegaly—3 (8.3), oesophageal dysmotility—3 (8.3), myositis—2 (5.6),
Anaya et al. [17]	–	–	–	3 (23.1)	13 (100)	12 (92.3)	6 (46.2)	3 (23.1)	1 (7.7)	Total—3 (23.1), CN—1 (7.7), PNS—1 (7,7), CNS—1 (7.7)	GN—1 (7.7)	–	Hepatitis—2 (15.4), non-Hodgkin’s lymphoma—2 (15.4), pancreatitis—1 (7.7), thyroiditis—1 (7.7), myositis—1 (7.7)
Horvath et al. [18]	–	–	–	–	60 (100)	–	41 (68.3)	4 (6.7)	0 (0.0)	–	GN/RTA—3 (5.0)	6 (10.0)	Vasculitis—9 (15.0), serositis—5 (8.3), myositis—5 (8.3)
Gondran et al. [19]	38 (90.5)	40 (95.2)	15 (35.7)	–	–	37 (88.1)	16 (38.1)	12 (28.6)	8 (19.0)	11 (26.2)	2 (4.8)	2 (4.8)	Dermatological—9 (21.4), lymphoma—4 (9.5), myositis—4 (9.5), depression—2 (4.8)
Cervera et al. [20]	18 (94.7)	18 (94.7)	3 (15.8)	–	–	10 (52.6)	4 (21.1)	0 (0.0)	3 (15.8)	PN—2 (10.5)	–	–	
Diaz-Lopez et al. [21]	–	–	5 (17.9)	–	–	–	8 (28.6)	5 (17.9)	–	–	–	–	Carpal tunnel syndrome—4 (14.3), fibromyalgia—2 (7.1), thyroidopathy—1 (3.6), liver disease—1 (3.6)
Drosos et al. [22]	–	–	2 (16.7)	–	9 (75.0)	–	1 (8.3)	1 (8.3)	–	–	–	4 (33.3)	Constitutional—4 (33.3), hepatomegaly—2 (16.7)
Paediatric studies													
Yokogowa et al. [23]	16 (61.5)	17 (65.4)	16 (61.5)	6/16 (37.5)	21 (80.8)	22 (84.6)	15 (57.7)	–	–	6 (23.1)	Total—5 (19.2), nephrocalcinosis—2 (7.7), RTA—2(7.7), nephritis—1 (3.8)	12 (46.2)	Dental caries—15 (57.7), fever—6 (23.1), haematological—4 (15.3), dermatological—4 (15.3), uveitis—2 (7.7)
Cimaz et al. [24]	5 (12.5)^c^	5 (12.5)^c^	29 (72.5)^c^	28 (70.0)	14 (35.0)^c^	–	4 (10.0)^c^	4 (10.0)^d^	–	2 (5.0)	RTA—3 (7.5)^d^	3 (7.5)^d^	Vulvovaginitis—5 (12.5), hepatitis—4 (10.0)^d^, fever—4 (10.0) ^c^/3 (7.5)^d^, fatigue—3 (7.5)^c^
			23 (57.5)^d^		25 (37.5)^d^								
Drosos et al. [22]	–	–	10 (76.9)	–	3 (23.1)	–	3 (23.1)	4 (30.8)	–	–	–	3 (23.1)	Constitutional—3 (23.1), hepatomegaly—1 (7.8)
Stiller et al. [25]	–	–	6 (54.5)	8 (72.7)	–	–	–	–	–	–	–	–	–
Tomita et al. [26]	–	–	15 (35.7)	15 (35.7)	10 (23.8)	21 (50.0)	11 (26.2)	0 (0.0)	1 (2.4)	Aseptic meningitis—3 (7.1)	Nephritis—3 (7.1)	11 (26.2)	Fever—22 (52.4), fatigue—12 (28.6), erythema—11 (26.2), hypothyroidism—2 (4.8), uveitis—2 (4.8), raised AST—1 (2.4), morning stiffness—1 (2.4)

*AST* aspartate Aminotransferase, *CN* cranial neuropathy, *CNS* central nervous system, *EG* extra-glandular, *GN* glomerulonephritis, *PN* peripheral neuropathy, *PNS* peripheral nervous system, *RTA* renal tubular acidosis

^a^Schirmer’s *I* test <5 mm/5 min

^b^Set by inclusion criteria

^c^At onset

^d^At follow-up

**Table 3:** Serological and histological features of pSS in male studies

References	Positive biopsy *N* (%)	Anti-SSA *N* (%)	Anti-SSB *N* (%)	ANA *N* (%)	RF *N* (%)	Cryoglobulinaemia *N* (%)	Other *N* (%)
Molina et al. [16]	29/31 (93.5)^a^	7 (19.4)	2 (5.6)	19 (52.8)	8 (22.2)	–	Raised Ig—7 (19.4)
Anaya et al. [17]	–	8 (61.5)	6 (46.2)	11 (84.6)	8/11 (72.7)	1/9 (11.1)	Lymphopenia—5 (38.5)
							Neutropenia—1 (7.7)
Horvath et al. [18]	–	45 (75.0)	31 (51.7)	22 (36.7)	16 (26.7)	4 (6.7)	Other ENA—37 (61.6)
							Anti-CCP—9 (15.0)
							dsDNA—4 (6.7)
							Anti-thyroglobulin—1 (1.7)
							Anti-TPO—1 (1.7)
Gondran et al. [19]	42 (100)^b^	21 (50.0)	14 (33.3)	32 (76.2)	22 (52.4)	8 (19.0)	Raised Ig—20 (47.6)
							Thrombocytopenia—9 (21.4)
							Lymphopenia—3 (7.1)
							Leukopenia—1 (2.4)
Cervera et al. [20]	–	3 (15.8)	3 (15.8)	13 (68.4)	5 (26.3)	1/14 (7.1)	Hypocomplementaemia—0/16 (0.0)
Diaz-Lopez et al. [21]	–	7 (25.0)	4 (14.3)	22 (78.6)	13 (46.4)	–	Raised IgA—11 (39.2)
							Raised IgG—7 (25.0)
							Raised IgM—2 (7.1)
							Anti-thyroid—2 (7.1)
							Low IgM—1 (3.5)
Drosos et al. [22]	–	2 (16.7)	1 (8.3)	4 (33.3)	8 (66.7)	–	–

*ANA* anti-nuclear antibody, *Anti-CCP* anti-cyclic citrullinated peptide, *Anti-TPO* anti-thyroid peroxidase, *dsDNA* double stranded DNA, *ENA* extractable nuclear antigen, *Ig* immunoglobulin, *TPO* thyroperoxidase, *RF* rheumatoid factor

^a^Grade III/IV Greenspan Criteria

^b^Criteria unknown

**Table 4:** Serological and histological features of pSS in paediatric studies

References	Abnormal sialometry *N* (%)	Abnormal salivary scintigraphy *N* (%)	Abnormal ocular stain *N* (%)	Positive biopsy *N* (%)	Positive sialography *N* (%)	Anti-SSA *N* (%)	Anti-SSB *N* (%)	Anti-SSA and/or anti-SSB *N* (%)	Anti-ENA *N* (%)	ANA *N* (%)	RF *N* (%)	Raised ESR *N* (%)	Raised IgG *N* (%)
Yokogowa et al. [23]	5/7 (71.4)^a^	3/3 (100)	4/16 (25.0)	10/15 (66.7)^b^ 15/15 (100)^c^	–	22/26 (84.6)	17/26 (65.4)	22 (84.6)	–	25 (96.2)	16 (72.7)	–	–
Cimaz et al. [24]	–	30 (75.0)	–	40 (100)^d^	40 (100)	–	–	–	29 (72.5)	34 (85.0)	30 (75.0)	27 (67.5)	21 (52.5)
Drosos et al. [22]	–	–	–	–	–	7 (53.8)	4 (30.8)	11 (84.6)	11 (84.6)	9 (69.2)	6 (46.2)	–	–
Stiller et al. [25]	9 (81.8)^e^	–	8 (72.7)	10 (90.9)^d^	10 (90.9)	4 (36.4)	3 (27.3)	7 (63.6)	–	7 (63.6)	3 (27.3)	–	2 (18.2)
Tomita et al. [26]	–	–	11 (26.2)	29 (69.0)^f^	31 (73.8)^g^	26 (61.9)	13 (31.0)	–	–	34 (81.0)	24 (57.1)	–	32 (76.2)

Anti-ENA- Anti-Extractable Nuclear Antigen; ANA- Anti-nuclear Antibody; ESR- Erythrocyte Sedimentation Rate; LSG FS- Labial Salivary Gland Focal Score; RF- Rheumatoid Factor

^a^<0.1 ml/min

^b^LSG FS >1

^c^LSG FS > 0

^d^Criteria unknown

^e^<0.02 ml/min

^f^Grade III/IV Greenspan Criteria

^g^Stage III/IV Rubin and Holt Criteria

### Assessment of study quality

We used a critical appraisal tool (The Joanna Briggs Institute Prevalence Critical Appraisal Tool) designed for use in systematic reviews addressing questions of prevalence to assess the quality of our studies [14]. The appraisal tool comprises nine assessment questions related to the quality of studies including the population representativeness, recruitment strategy, sample size, description of study subjects, use of objective, standard criteria for subject classification, quality of outcome measurements, appropriate statistical analysis, identification of confounding factors and identification of subpopulations, where appropriate. These criteria were assessed as present, absent, unclear or not applicable, as per the methodological guidance published for this critical appraisal tool [15]. The assessment was completed by all three authors independently and a consensus was reached for every study. Because of the low prevalence of pSS in the paediatric and male populations, the majority of the studies evaluated cohorts from secondary and tertiary centres and used various classification criteria (justified by the lack of classification criteria in the paediatric population, and various revisions of classification criteria for adult pSS over time). In addition, the measurement of different clinical manifestations was not standardised and it relied on medical records and expert opinion. The data analysis did not have significant coverage of the identified sample as, in the majority of studies, the paediatric cases were not compared with adult patients, and the male case was pre-selected and compared to consecutive female case series from a different cohort.

A compromise was made by the authors to include papers which used different classification criteria for pSS because of the small number of studies available in the male and paediatric populations. Consequently, and in addition to the lack of fulfilment of the majority of the nine questions included in the appraisal checklist and because of the high study heterogeneity, we could not calculate reliable pooled prevalence rates for different disease manifestations and perform a meta-analysis.

## Results

### Demographics/epidemiological

After assessment of study quality, we included data from seven studies on the male population [16–22] and five on the paediatric population [22–26]; this data is presented in Table 1. The total adult male cohort included in our systematic review consisted of 210 patients (range 12–60 patients/per study). The paediatric cohort consisted of 132 patients (range 11–42 patients/per study). All of the male papers included comparisons with female patient population, while in the paediatric studies, only one study compared children with adult pSS population [23]. Three studies reported on patients from the American population, eight were European and one was Japanese. The criteria for patient inclusion varied. In the male studies, four papers used the European Community Study Group (ECSG) classification [27], two papers used the 2002 AECG [28] and one paper used its own personal criteria (Table 1). One paediatric study used the ECSG criteria, one used the European League Against Rheumatism (EULAR) criteria [29] and the other three studies used different personal criteria. The mean age of the patients was available for all studies. However, three different data sets were described: age at onset, age at presentation and age at diagnosis. Four out of the five paediatric papers included in our final analysis quoted age at onset (range 9.5–10.7 years). Across the cohort of paediatric patients, where reported, the female:male ratio was 8:1.

### Clinical features

The data regarding clinical features are presented in Table 2.

#### Glandular features

Data about the prevalence of parotitis were available in 10 out of 12 studies. A total of 35 adult male and 76 paediatric patients presented with parotitis as a manifestation of pSS (range 15.8–35.7% and 35.7–76.9%, respectively). All studies reported on dryness, although this was classified as ‘any sicca’ symptoms in some studies and as ‘dry eyes’ and ‘dry mouth’ in others. In the four male studies that reported sicca symptoms, these were present in 75.0–100% of patients. In the four paediatric studies reporting dryness, the subjective sicca symptoms were present in 23.1– 80.8% of patients.

#### Extra-glandular features

The presence of any extra-glandular features was described in four papers reporting on the male population and in two of the paediatric papers. A proportion of 52.6–92.3% of male patients and 50.0–84.6% of paediatric patients had these manifestations. Joint involvement was the most commonly ascertained data set (all studies, apart from Stiller et al. 2000 ( [25]) reported on it). Joint involvement, defined as arthritis or arthralgia, was reported in 8.3– 77.8% of male patients and 10.0–57.7% of paediatric patients. Only two papers reported separately on arthritis and arthralgia in male population with pSS, arthralgia being found in 18/28 and 5/6 of patients with joint involvement, respectively [16, 17]. Raynaud’s, pulmonary and neurological involvement were reported in both the male (8.3–28.6%, 0.0–19.8% and 10.5–38.9%, respectively) and paediatric populations (0.0–30.8%, 2.4% and 5.0–23.1%, respectively). Renal involvement and lymphadenopathy were found in children in the following proportions in different studies: 7.1–19.2% and 7.5–46.2%, respectively, while the frequency of these symptoms was lower in the male population (2.8–7.7% and 4.8–33.3%, respectively). Many other extra-glandular features were occasionally reported; of note, there was a high prevalence of fever (52.4%) and dental caries (57.7%) reported by two paediatric studies [23, 26].

### Laboratory features

Due to variation of the laboratory features reported between the male and paediatric studies, the data has been presented separately (in Tables 3 and 4). With the exception of one paper, which reported on the presence of anti-extractable nuclear antigen (ENA) antibodies [24], all papers were reported on the core serology—the presence of anti-SSA (Ro), anti-SSB (La), anti-nuclear antibodies (ANA) and rheumatoid factor (RF). Anti-SSA, anti-SSB, ANA and RF were positive in 15.7–75.0%, 5.6–51.7%, 33.3–84.6%, 22.2–72.7%, respectively, in the male population, and 36.4–84.6%, 27.3–65.4%, 63.6–96.2%, 27.3–75.0%, respectively, in the paediatric population, suggesting a high variability between different studies. Cryoglobulinaemia was only reported in the male population (in four out of seven studies) with a prevalence of 6.7–19.0%. A number of other serological markers were occasionally reported (Tables 3 and 4).

Data on non-serological investigations were available for the paediatric population and included pathological biopsy, sialometry, scintigraphy, ocular stains and sialography. In the male population, only salivary gland biopsy data were retrievable from the papers included in our analysis, with a proportion of 93.5–100% patients having a positive biopsy. In the paediatric population, a pathological biopsy ranged from 66.7 to 100%; two out of the four studies described no criteria for defining the histological criteria used for pSS classification.

In Table 5 (supplementary information), we summarised the main differences between male and female populations, and paediatric and adult populations as reported by the studies included in our analysis; however, comparisons between this systematic review and data available from other systematic reviews in adult pSS populations were beyond the purpose of this paper.

## Discussion

Although pSS is an autoimmune disease with well-recognised female predomination, a proportion of up to 10% of adult patients diagnosed with this condition are male. In the paediatric population, the female to male ratio is 6–7:1 [24, 30]. The diagnosis of pSS in children is delayed, in many cases, because children less frequently report dryness due to their good saliva and tear reserve and frequently present with extra-glandular clinical features suggestive of other autoimmune diseases [31]. Although the disease burden is significant in both male and paediatric populations, good quality studies are lacking. To our knowledge, this is the first attempt to review systematically the literature on male and paediatric populations with pSS.

Many of the studies we selected based on our inclusion criteria involved the selection of relevant paediatric or male cases from large cohorts.

The classification criteria for pSS were revised several times in the last few years and again very recently [11, 12]; therefore, different inclusion criteria were used in different studies. The main difference between the ECSG [29] and AECG [28] classification criteria is that the AECG criteria require the presence of positive biopsy or serology tests to classify patients as having pSS. In addition, the AECG criteria also mention the need to exclude mimicking pathology, such as head and neck radiation treatment, hepatitis C and use of anticholinergic drugs.

We have taken into consideration that a comparison between studies including patients classified in different ways might lead to erroneous conclusions; therefore, we presented the results as intervals of proportions of patients reported as having different disease manifestations. A pooled prevalence meta-analysis could not be performed because of the high heterogeneity of the patient population, which would have led to unreliable conclusions.

A significant proportion of studies (5/12) included a low number of patients (less than 20). Although all the male studies also evaluated a certain number of female patients, the male cases were pre-selected (because of their rarity) and compared to a variable number of consecutive female cases (therefore, all the studies were affected by patient selection bias).

One of the other limitations of this systematic analysis was the selection of paediatric studies which relied on expert opinion for diagnosis, which is the consequence of lack of specific classification criteria for paediatric pSS. It is recognised that the available classification criteria designed for adult pSS population have significant limitations when used in children [32].

One of the main conclusions of this systematic analysis was that parotid gland involvement was common in children with pSS despite being recognised that, with the exception of mumps parotitis, all other causes of parotitis in children are rare [33]. Parotitis is therefore recognised as a characteristic disease feature in paediatric pSS [31]. Sicca symptoms were less common in children for the reasons discussed above. Symptoms of dryness are recognised to be more common in the adult population affected by pSS because of the correlation between the destruction of the acinar cells and age [34, 35]. Despite this, the symptoms of eye dryness were reported with a high variability in the male studies, with a prevalence range of 38.9–94.7%.

We also found that in the paediatric population, constitutional symptoms and lymphadenopathy were prevalent, which can be explained by a particular disease phenotype in children or other confounders (frequent associated viral infection during childhood, parental vigilance in detecting high temperature, etc.). However, lymphadenopathy despite being generally less commonly reported in the adult population, it was found as more common in male than female patients in only one study [18]. The joint involvement in adult pSS, partly explained by the disease phenotype and partly by the age-related associated joint pathology, showed no specific trend in the male versus female population, as the studies included in our analysis reported inconsistent data ( [18], [20], [22]).

There was a significant variation of positive serological markers in both the male and paediatric populations. As the quality of studies and patient classification criteria used were very heterogeneous, we cannot infer any reliable comparison with the reported positive serology prevalence from pSS adult studies [36]. The trend towards a higher positive serology prevalence in children can be explained again by the difficulty to diagnose pSS in children with reasonably well-preserved exocrine gland secretion without specific serology, and could explain the need for additional investigations to facilitate the diagnosis (we found that invasive investigations, such as scintigraphy, sialometry and biopsy were more frequently performed in paediatric studies). It was previously identified that the presence of antibodies directed against the Ro/La ribonucleoprotein complexes have been correlated with younger age, more severe dysfunction of the exocrine glands and a higher prevalence of extra-glandular manifestations in the adult population [36]. Our systematic analysis identified a prevalence of extra-glandular manifestations between 52.6–92.3% in the male population and 50.0–84.6% in children, while abnormal sialometry was reported only in the paediatric population, with a prevalence between 71.4 and 81.8%.

A case series of children with pSS reported that although the serological markers were not relevant for diagnosis, all patients had specific lymphocytic infiltration of labial salivary glands and sialectasis [37]; subsequently, the authors concluded that salivary and lachrymal gland histopathology in this age group is highly recommended for accurate diagnosis.

Previous research comparing pSS patients with early and late disease onset found no significant differences between their clinical presentation and incidence of diagnostic test positivity; however, the early onset of pSS was defined as diagnosis before the age of 40 and the study did not include children [5]. A recent systematic review and meta-analysis of pSS epidemiology reported a female/male ratio in prevalence rate for pSS of 10.72:1, without providing details about the differences in clinical presentation or laboratory features of pSS in female compared to male patients [38].

In summary, this systematic review found that children diagnosed with pSS reported less frequently symptoms of dryness, and had a higher prevalence of systemic symptoms, including fever and lymphadenopathy, as well as parotitis, in comparison to adult populations [39, 40].

Male patients with pSS were younger at the time of diagnosis than the pooled aged at diagnosis in the general pSS adult population, as estimated by a recent systematic review and meta-analysis [38]; however, male patients did not have any consistently different clinical or serological features when compared to female pSS patients in different studies, which can be explained by different pSS classification criteria used, patient selection bias or true heterogeneity of the male pSS population.

Our systematic review highlighted the difficulties related to data collection in rare populations. In addition, the different clinical presentation and absence of validated classification criteria for the paediatric population with pSS in comparison to the adult population makes reliable diagnosis very difficult and highly dependent on expert opinion.

We also found that despite the increased clinician interest in defining different disease phenotypes, such as male and paediatric pSS, all the studies available came from developed countries and their conclusions are difficult to extrapolate.

In conclusion, our systematic review highlighted the most commonly reported clinical manifestations and serological markers of pSS in male and children population, raising clinicians’ awareness about particular disease features in populations rarely affected by pSS. This could have significant implications in facilitating the diagnosis of pSS in male and children. Future work, including large longitudinal cohort studies comparing male versus female patients, and adult versus paediatric patients, will enable us to differentiate reliably the disease presentation and evolution in these selected categories of patients.

### Significance and innovation

- This is the first systematic review of pSS in rare populations
- Large, multicentre, good-quality studies of pSS in rare populations and classification criteria for pSS in paediatric population are lacking
- Children with pSS have less dryness, and more frequently experienced systemic symptoms and parotid enlargement
- There were no consistent differential features of male pSS patients when compared to adult female patient population

## Electronic supplementary material

Table 5 (DOCX 20 kb)
