# Supplementary material for: A systematic review of primary Sjögren’s syndrome in male and paediatric populations
Source: Clin Rheumatol. 2017 Jul 22;36(10):2225–36. doi: 10.1007/s10067-017-3745-z (PMC5596040; doi:10.1007/s10067-017-3745-z)
Supplement: Supplementary file 1 — (DOCX 20 kb) [file 10067_2017_3745_MOESM1_ESM.docx]

**Table 5:** Comparison between male and female pSS patients and paediatric and adult populations based on the studies included in the systematic review.

| ***Male studies*** | | |
| --- | --- | --- |
| **References** | **Male features** | **Female features** |
| **Molina et al. 1985 (16)** | Less RF, anti Ro antibodies,  HLA-B8, HLA-DR3 frequency similar to normal controls | HLA- Dw52 more frequent than in normal controls |
| **Anaya et al. 1995 (17)** | No significant differences | |
| **Horvath et al. 2008 (18)** | More arthritis, vasculitis, lymphadenopathy | More Raynaud’s phenomenon and thyroiditis |
| **Gondran et al. 2008 (19)** | Less depression and asthenia  More thrombocytopaenia and increased risk of lymphoma | More fibromylagia |
| **Cervera et al. 2009 (20)** | Less arthritis | No other differences |
| **Diaz-Lopez et al. 2004 (21)** | Increased Ig A, RF, ANA antibodies |  |
| **Drosos et al. 1997 (22)** | Less arthritis, less ANA and anti Ro antibodies | More Raynaud’s phenomenon |
| ***Paediatric studies*** | | |
| **References** | **Paediatric features** | **Adult features** |
| **Yokogowa et al. 2016 (23)** | More parotitis, positive serology, neurologic and renal manifestations, and non-specific features (fever, lymphadenopathy)  Less commonly had symptoms of dry mouth and dry eyes | More adult patients than children fulfilled the classification criteria for pSS |
| **Cimaz et al. 2003 (24)** | Frequent recurrent parotid swelling  Sicca symptoms rare | No comparison available |
| **Drosos et al. 1997 (22)** | Similar immunological profile | |
| **Stiller et al. 2000 (25)** | No comparison available | |
| **Tomiita et al. 1997 (26)** | Frequent systemic manifestations (fever, exanthema, arthralgia, etc.) except for sicca symptoms at onset.  Serological markers of pSS frequently observed | No comparison available |
| ANA- Anti-Nuclear Antibody; Ig- immunoglobulin, pSS- primary Sjӧgren’s syndrome; RF- Rheumatoid Factor | | |
